# RETRACTED: Effect of a Hemodialysis Session on Markers of Inflammation and Endotoxin

**DOI:** 10.1155/ijin/9806786

**Published:** 2026-11-14

**Authors:** International Journal of Inflammation

RETRACTION: S. Dheda, D. A. Vesey, C. Hawley, D. W. Johnson, and M. Fahim, “Effect of a Hemodialysis Session on Markers of Inflammation and Endotoxin,” *International Journal of Inflammation* 2022, no. 1 (2022): 1–7, https://doi.org/10.1155/2022/8632245.

The above article, published online on 10 March 2022 in Wiley Online Library (https://wileyonlinelibrary.com), has been retracted by agreement between the authors; the journal Editor, Satabdi Datta Choudhury; and John Wiley & Sons Ltd.

The retraction has been agreed following a request from the authors, who identified issues regarding the consent and ethics concerning the control group participants. The authors confirmed that verbal consent was obtained but had not been appropriately documented. Additionally, it was discovered that the protocol reviewed by the Cairns and Hinterland Human Research Ethics Committee had not described the inclusion of the control group.

Due to these oversights, the authors notified the responsible ethics committee who subsequently requested the retraction of the article. On review of the information provided by the authors, the article is hereby retracted.

